# Influence of Boron Additions and Heat Treatments on the Fatigue Resistance of CoCrMo Alloys

**DOI:** 10.3390/ma12071076

**Published:** 2019-04-01

**Authors:** Marco A. L. Hernandez-Rodriguez, Rafael D. Mercado-Solis, Gerardo Presbítero, Diego E. Lozano, Gabriela M. Martinez-Cazares, Yaneth Bedolla-Gil

**Affiliations:** 1 Facultad de Ingeniería Mecánica y Eléctrica de la Universidad Autónoma de Nuevo León, Av. Universidad S/N Ciudad Universitaria, San Nicolás de los Garza CP 66451, Mexico; malhdz@gmail.com (M.A.L.H.-R.); rafael.mercadosl@gmail.com (R.D.M.-S.); 2 Facultad de Ingeniería, Universidad Nacional Autónoma de México, Polo Universitario de Tecnología Avanzada (PUNTA/UNAM), Monterrey 66629, Mexico; gpresbitero@yahoo.com.mx; 3 Departamento de Ingeniería, Universidad de Monterrey, Av.Morones Prieto 4500, San Pedro Garza García 66238, Mexico; diego.lozanod@udem.edu (D.E.L.); gabriela.martinezc@udem.edu (G.M.M.-C.)

**Keywords:** CoCrMo, fatigue strength, boron, heat treatment, biomaterial, fatigue crack growth

## Abstract

Cobalt-based alloys are widely used in the manufacture of joint prostheses. In this study, the effect of boron additions and heat treatment on the ASTM F75 was evaluated by rotating bending fatigue. The boron ranged from 0.06–1 wt %. The alloys were tested in as-cast and heat-treated conditions. In the as-cast condition, the infinite life was observed at 380 MPa, improving to 433–615 MPa according to the amount of boron added. In the heat treatment condition, the fatigue resistance was improved only in the base alloy. The addition of 0.06 wt % boron and heat treatment led to the same resistance as in the as-cast condition. Adding large amounts of boron combined with heat treatment diminished the fatigue limit. The fracture analysis revealed primarily brittle behaviour with some ductile features even on the same sample; only the heat-treated alloy with 0.06 wt % boron was clearly ductile. This alloy also exhibited notably better toughness to crack propagation.

## 1. Introduction

Nowadays, the standard treatment for degenerative joints is to replace them with medical devices, such as knee and hip prostheses, 70–80% of which are made of metallic biomaterials [1]. Such devices face severe environmental conditions. The concentrations of chloride ions in the serum and the interstitial fluid are 113 and 117 mEq L^−1^, respectively, while the oxygen dissolved in blood is one-fourth of that in air [2]. Also, the pH in body fluids decreases to 5.2 when a material is implanted in the hard tissue and takes about two weeks to recover to 7.4 [3]. In addition, metallic biomaterials have to fulfil strict requirements. The main metallic biomaterials are stainless steels, cobalt–chrome alloys, and titanium alloys, each with their own advantages and disadvantages. Titanium alloys present a lower elasticity modulus compared to stainless steel and Co–Cr alloys, which reduce the stress shielding effect [4,5]. The effect of different alloying elements on the microstructure of titanium alloys and their elastic modulus has been reported [6,7,8]. However, titanium implants are fatigue damaged due to wearing and fretting, which leads to corrosion pits on the surface and consequently a decrease in fatigue resistance [4]. Cobalt alloys exhibit high corrosion resistance and excellent wear resistance [1]. The clinical use of these alloys for long periods of time has shown good biocompatibility in bulk form [2]. However, the fatigue process assisted by corrosion has been reported as the failure mechanism of cobalt–chromium–molybdenum (CoCrMo) orthopaedic devices [3]. The alloy ASTM F799, i.e., the wrought version of ASTM F75, is currently the most frequently used alloy in structural materials for permanent implants at load-bearing sites, such as artificial joints, and is intended to provide at least 20 years of service [9]. However, these implants experience severe cyclic loading. The fatigue performance of cast and forged Co alloys decreases in simulated body fluids, which together can be the cause of the low success of total hip replacements after 20 years [9]. This has led to exhaustive research to improve the performance of these devices, particularly when considering young, active patients.

Fatigue fractures occur because of stress concentration near certain geometrical sites. The maximum tensile stresses in the stem and neck of a hip prosthesis are approximately 200 and 350 MPa, respectively [9]. The fatigue limit of implantable alloys depends on factors such as the fabrication process, surface conditions, microstructure, and fatigue conditions [10]. The fatigue resistance of forged alloys is considerably higher than that of cast alloys; for example, the fatigue strength of forged Co–Cr exceeds 600 MPa (in air) compared with 300 MPa of cast Co–Cr [9,10,11]. Forged Co–Cr has higher fatigue strength due to the wrought microstructure obtained via solid-state phase transformation from a face-centred cubic to a hexagonal close-packed (HCP) crystal structure during cold working [9,12]; therefore, the fatigue, yield and ultimate tensile strengths of the F799 alloy are approximately twice those of as-cast ASTM F75 [9]. However, for the alloy to be forged easily, its carbon content must be sufficiently low, which compromises the wear resistance [9,12]. Meanwhile, because of the brittle nature of the HCP structure, the generation and propagation of cracks are generally accelerated [12].

Interstitial nitrogen in a low-carbon (0.14 wt %) Co–Cr alloy was shown to increase the yield strength while maintaining good ductility; however, this effect was not observed in fatigue tests possibly because of flaws within the test pieces [13]. The fatigue resistance of Co-based Vitallium 2000 Plus casting alloy increased upon the addition of 0.1 wt % N; microvoids and coalescence were observed at the fatigue surface fracture which are typical characteristics of a ductile material [14]. In a heat-resistant Co-based alloy with 0.09–1.48 wt % boron, a sensitive reduction was observed in the incubation time of carbides. Moreover, adding small amounts of boron refined the grain size of the primary phases, whereas adding large amounts led to the formation of metallic borides [15]. Zhuang and Langer [16] reported improved mechanical properties, particularly ductility, for a cobalt–chromium–molybdenum (CoCrMo) alloy with nickel and traces of aluminium, titanium and boron. This alloy also exhibited improved resistance to the growth of fatigue cracks due to the presence of Ni and the elimination of some microstructural casting defects by trace elements [17].

In contrast, microstructural alterations due to heat treatment influence the mechanical behaviour of materials under static and cyclic stresses. Therefore, the fatigue behaviour of Co-based implants can be improved by controlling the alloy composition and via heat treatment [18]. Dobbs and Robertson [19] found the lamellar constituent to be the most detrimental phase in the microstructure of Co–Cr alloys; the removal of this phase via heat treatment improved the fatigue behaviour.

The effect of boron in CoCrMo nickel-free alloys has not been reported in the literature. Therefore, this study aims to develop new Co-based alloys with added B as a potential material for medical applications in the replacement of joints damaged by severe trauma and arthritis. The specific objective of this study is to assess the fatigue performance of these alloys under both as-cast and heat-treated conditions. It is worth mentioning that these alloys were also evaluated in corrosion resistance [20].

## 2. Materials and Methods

### 2.1. Materials

Co-based alloys were obtained via investment casting, which allowed the manufacture of semi-finished shapes in ceramic moulds. The chemical composition specified in ASTM F75 was used as a reference and compared with boron additions in the amounts of 0.06, 0.25, 0.5 and 1 wt % (Table 1). The specimens were tested in the as-cast and after heat treatment conditions. The heat treatment was performed at 1200 °C for 1 h followed by water quenching. The temperature for the heat treatment was chosen from the results observed in previous works [21,22]. Table 2 identifies the various alloys.

### 2.2. Specimens and Equipment

**Figure 1:**
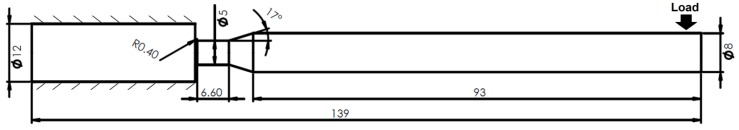
Dimensions of samples evaluated in bending fatigue tests (mm).

Fatigue samples were machined at the dimensions required for evaluation in the rotating bending fatigue equipment described in a previous work [23]. The loading was applied by constant deflection, implying that the load decreased as the crack grew. Figure 1 shows the geometry and dimensions of the fatigue specimens.

### 2.3. Stress and Stress Concentration Factor

**Figure 2:**
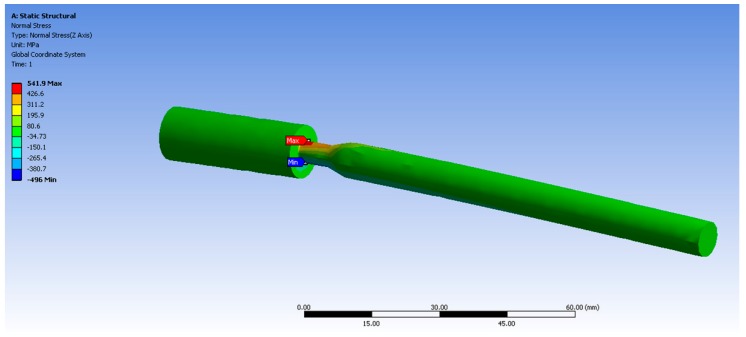
Normal stress (MPa) on the fatigue sample for a load of 50 N.

The evaluated fatigue samples had a stress concentration to promote the fracture in a controlled zone. The effect of a notch on the local stress depends on the geometry and root radius; for this specific part in bending, the stress concentration factor (*K*_t_) was determined as follows [24]:

(1) $$K_{t}=\frac{\sigma_{\max}}{\sigma_{\mathrm{nom}}}$$

where σ_nom_ is the nominal stress and σ_max_ is the maximum local elastic stress acting at the notch. The nominal stress at the surface of a bending shaft is calculated as [24]:

(2) $$\sigma_{\mathrm{nom}}=\frac{32M}{\pi d^{3}}$$

where *M* is the bending moment and *d* is the smaller diameter of the sample. The σ_max_ was determined by finite element analysis (FEA) in the ANSYS Workbench package (version 13.0, USA) with 3210 nodes and 1794 elements; the estimated stresses are shown in Figure 2. Using the σ_max_ obtained via FEA and the σ_nom_ computed using Equation (2), a *K*_t_ = 5.4 was obtained from Equation (1). Thus, the reported stresses are the values of σ_max_ calculated using this *K*_t_ value.

### 2.4. Experimental Procedure

The fatigue tests were performed in cantilever rotating bending with constant deflection at a frequency of 20 Hz and a stress ratio of *R* = −1. To construct the S-N curves (stress–number of cycles), the experiments were repeated three times at each stress level for each material condition. It was considered an infinite life at 10^7^ cycles. During testing, the load data were acquired to consider how the load decreases with macroscopic crack growth. Fracture surface analysis was carried out by means of scanning electron microscopy (SEM) and energy dispersive X-ray spectroscopy (EDS) in a JEOL JSM-6510LV (Tokyo, Japan).

## 3. Results

### 3.1. S-N Curves

**Figure 3:**
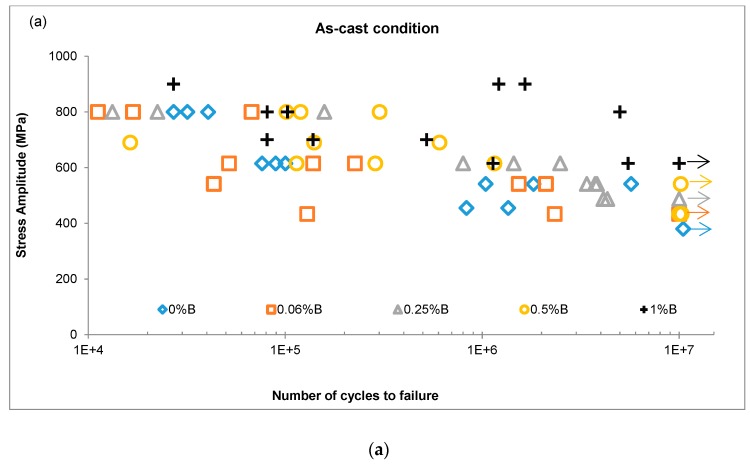

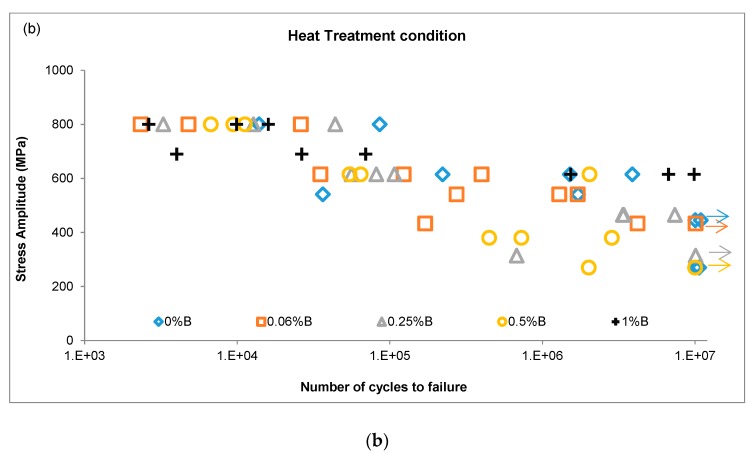
S-N diagrams of (**a**) as-cast and (**b**) heat treatment condition.

The S-N curves for the as-cast and heat-treated conditions are shown in Figure 3. A noticeable scatter of results is observed for the same alloy at the same stress level due to factors such as the microstructure and embedding of particles from the ceramic mould in the samples. Less dispersion could be expected if the manufacturing process were controlled more carefully.

The as-cast condition showed an improvement in the fatigue resistance with the addition of boron. The base alloy in this condition showed an infinite fatigue life at 380 MPa, whereas the alloys modified with boron exhibited increased resistance of 433, 487, 541 and 615 MPa according to the 0.06, 0.25, 0.5 and 1 wt % B content, respectively. The maximum fatigue resistance was observed at 615 MPa in the alloy with 1 wt % B, which is higher than the 300 MPa reported previously for the as-cast condition [11,19]. Sudhakar and Wang [25] reported an endurance limit of 387 MPa for a Co alloy with Ni additions. However, the use of Ni is limited in medical applications because of allergic reactions; ASTM F75-07 alloy restricts the Ni content to 0.5 wt % [26].

In contrast, among the heat-treated alloys, only the base alloy exhibited improved fatigue resistance compared with the as-cast condition, recording a value of 445 MPa. The fatigue limit for the 0.06B-HT alloy was 433 MPa, the same as that in the as-cast condition. The 0.25%B-HT and 0.5%B-HT alloys exhibited a fatigue resistance of 314 and 270 MPa, respectively. Regarding the 1%B-HT alloy, one sample withstood 9.85 million cycles at 615 MPa of stress amplitude.

The load data recorded during the fatigue tests allowed an estimation of the number of cycles occurring between the load drop and final fracture, which is related to the propagation rate of macroscopic cracks. Table 3 presents representative examples for each material evaluated regarding the approximate number of elapsed cycles in the final stage of the tests. In this case, the 0.06B-HT alloy displayed the best performance by enduring significantly more cycles than any other alloy before completing the failure, whereas the 1B-HT alloy exhibited the fastest crack propagation. The higher resistance to crack growth of the 0.06B-HT alloy could be due to the combined effect of (i) reducing microstructural casting defects by adding a small amount of B, as reported by Zhuang, with trace elements [17] and (ii) removing the detrimental phases by heat treatment, as described by Dobbs and Robertson [19]. In contrast, adding larger amounts of B increased the precipitation of boron carbides, leading to the alloys being more brittle and cracks usually propagating faster since the materials were more brittle.

### 3.2. Fatigue Crack Surfaces

**Figure 4:**
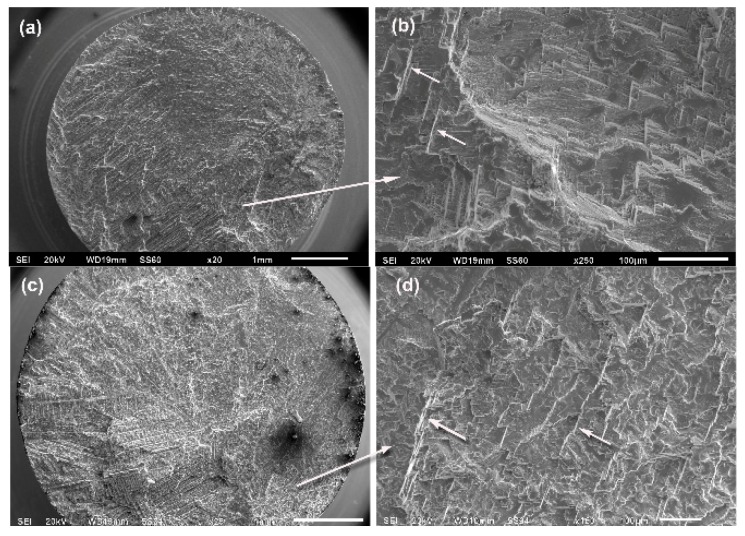
Fracture surfaces of alloys without B (arrows indicate striations): (**a**,**b**) 0B-AC alloy after 1,041,310 cycles at 541 MPa; (**c**,**d**) 0B-HT alloy after 3,879,407 cycles at 615 MPa; non-metallic inclusions can be seen in (**c**).

Analysing the fracture surfaces revealed mainly brittle behaviour; however, ductile features were also observed on the same sample. This could possibly be due to an increase in the strain-induced HCP phase after cyclic loading and a small amount of plastic strain after fracture due to the phase transformation [12]. Gueler [12] also stated that the amount of phase transformation was smaller with higher carbon content and smaller grain size. Figure 4 shows the brittle fracture surfaces in alloys without B, along with facets and striations. The fracture observed for the 0B-HT condition exhibited a high concentration of non-metallic inclusions from the ceramic mould, which act as crack generators.

**Figure 5:**
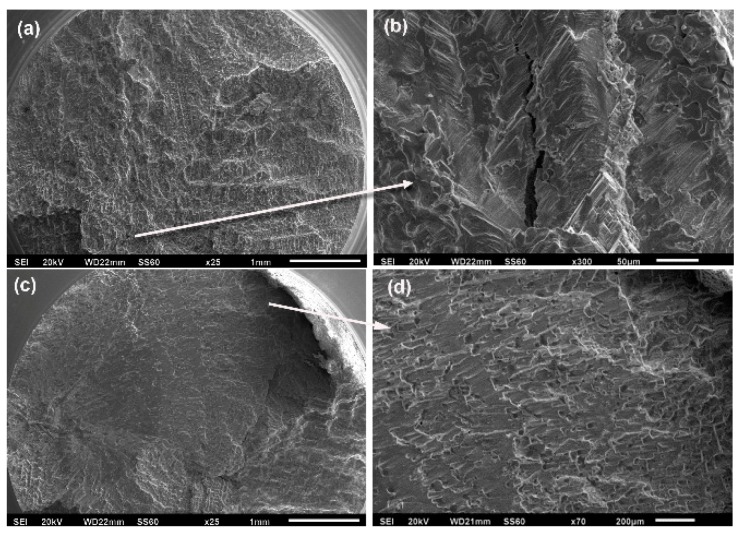
Fracture surfaces in alloys with 0.06 wt % B at 615 MPa: (**a**,**b**) 0.06B-AC alloy after 51,888 cycles; (**c**,**d**) 0.06B-HT alloy after 398,440 cycles.

Figure 5 shows the fracture surfaces of alloys with 0.06 wt % B. The 0.06B-AC alloy exhibited a transgranular fracture with microcracks and smoother reliefs, whereas the 0.06B-HT alloy exhibited a more ductile fracture with plastic deformation and dimples. The highest resistance to crack growth was also observed under this material condition. In a previous study, this alloy also showed good corrosion resistance [20]. The dark areas in some overview pictures were coloured with ink to identify certain areas during observations using scanning electron microscopy.

**Figure 6:**
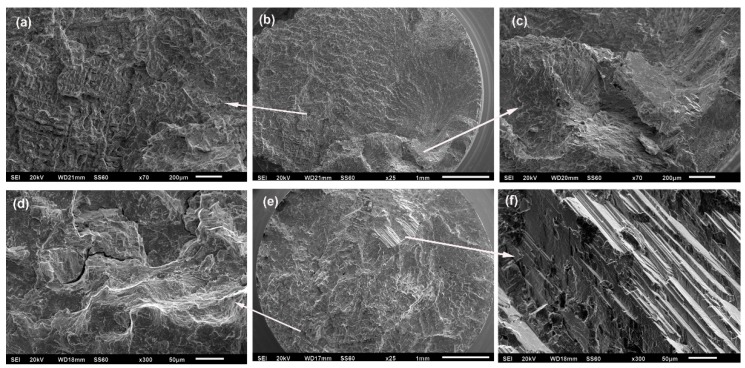
Fracture surfaces in alloys with 0.25 wt % B: (**a**–**c**) 0.25B-AC alloy after 3,738,217 cycles at 541 MPa; (**d**–**f**) 0.25B-HT alloy after 2,033,481 cycles at 615 MPa.

Figure 6 shows the fracture surfaces of alloys with 0.25 wt % B. The 0.25B-AC alloy exhibited a transgranular crack at the beginning that propagated in a radial direction, with dimples in the propagation zone and an intergranular final fracture. The 0.25B-HT alloy exhibited zones of faceted ‘stair-like’ deformation (brittle) and zones with dimples and microvoids (ductile).

**Figure 7:**
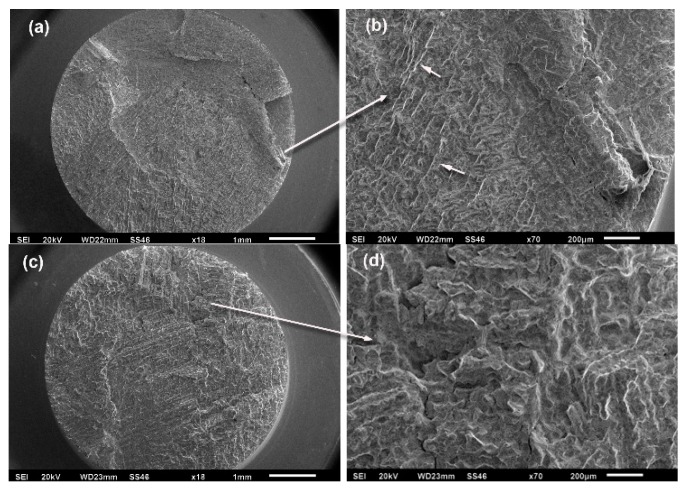
Surface fractures in alloys with 0.5 wt % B: (**a**,**b**) 0.5B-AC alloy after 4,196,143 cycles at 433 MPa; (**c**,**d**) 0.5B-HT alloy after 2,033,481 cycles at 615 MPa.

The fracture surfaces of alloys with 0.5 wt % B are shown in Figure 7. The 0.5B-AC alloy exhibited brittle striations that originated from the martensitic transformation induced by cyclic deformation at high crack growth [12,17]. The 0.5-HT alloy had an intergranular appearance at crack onset and during propagation and a transgranular appearance at the end.

**Figure 8:**
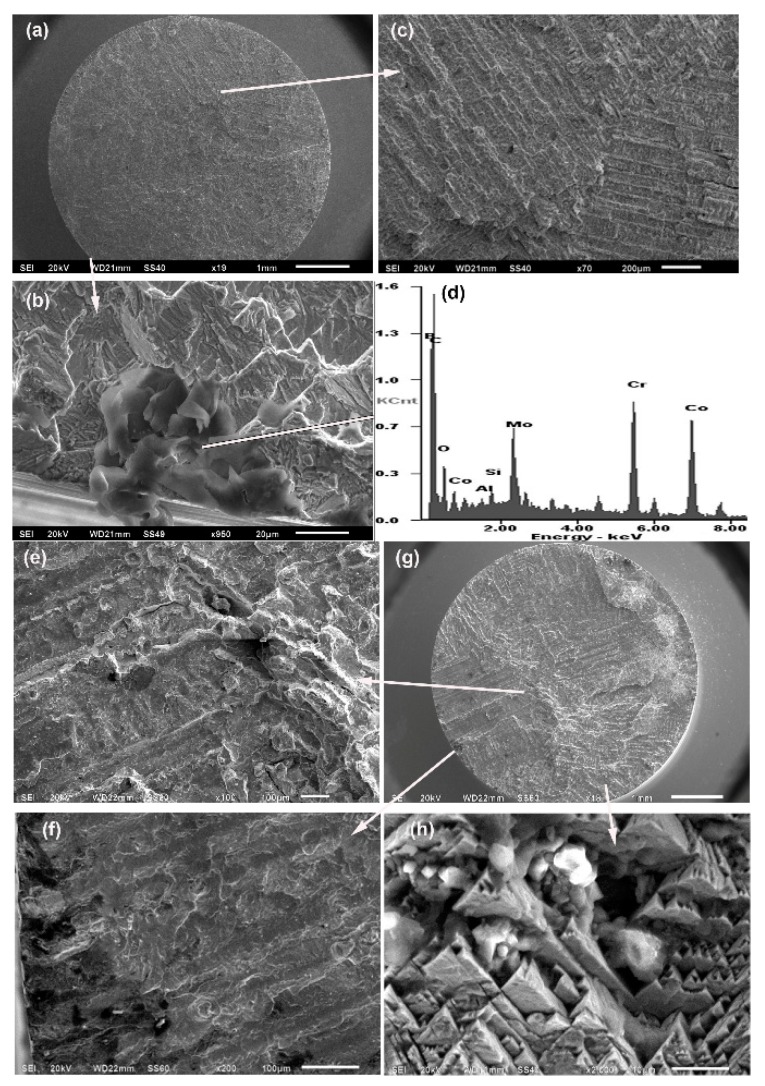
Crack surfaces in alloys with 1 wt % B: (**a**–**d**) 1B-AC after 522,944 cycles at 700 MPa; (**e**–**h**) 1B-HT after 9,849,976 cycles at 615 MPa.

The fracture surfaces in alloys with 1 wt % B are shown in Figure 8. The analysed sample of 1B-AC alloy exhibited a transgranular crack at the beginning and in the propagation zone and intergranular appearance at the end; the crack nucleated on a boron carbide. The 1B-HT alloy was the most brittle among the studied alloys; a stair-like fracture surface is observed with pyramids and cleavages. The pyramids arose from the intersection of three planes in a secondary cleavage crack [27]. The fracture in this 1B-HT sample originated on flaws generated by ceramic inclusions from the casting mould at the subsurface of the part. Nevertheless, the 1B-HT sample remarkably resisted 9.85 million cycles at 615 MPa before experiencing failure.

## 4. Conclusions

Modifying the chemical composition of Co-based alloys via the addition of B increased the fatigue resistance in the as-cast condition according to the amount of B added; infinite fatigue life of 615 MPa was observed for the alloy with 1 wt % B; this represents an improvement of 60% regarding the base alloy. In contrast, heat treatment diminished the fatigue resistance of most alloys containing B, except the 0.06B-HT alloy, which retained its fatigue resistance at the same level of 433 MPa as observed in the as-cast condition. On analysing the fracture surfaces, the 0.06B-HT alloy exhibited greater ductility compared with the brittle behaviour observed in the other alloys even when ductile features were observed in the same samples. The 0.06B-HT alloy also exhibited a remarkable toughness to crack propagation; it endured considerably more cycles compared with any other alloy from the onset of macroscopic cracks until the final fracture.

## Figures and Tables

**Table 1:** Chemical composition of the studied alloys (wt %).

Cr	Mo	C	Si	Fe	B	Co
27–30	8–11	0.08–0.26	0.17–0.74	0.3–0.8	0–1	balance

**Table 2:** Identification of materials.

%B	0	0.06	0.25	0.5	1
As-cast	0B-AC	0.06B-AC	0.25B-AC	0.5B-AC	1B-AC
Heat treated	0B-HT	0.06B-HT	0.25B-HT	0.5B-HT	1B-HT

**Table 3:** Number of cycles to failure (*N*) and approximate number of cycles during macroscopic crack propagation (*NCP*) at 615 MPa.

Material	As-Cast		Heat Treated	
	*N*	*NCP*	*N*	*NCP*
0B	100,120	7400	3,879,407	2500
0.06B	226,129	2700	398,440	31,500
0.25B	799,846	7900	107,191	3000
0.5B	1,153,988	3200	2,033,481	1600
1B	5,507,500	2900	9,849,976	900
